# Metabolic Disruption and Steatosis Induced by Drinking Water Disinfection Byproducts in HepG2 and HUH7 Cells

**DOI:** 10.3390/toxics14030269

**Published:** 2026-03-21

**Authors:** Marta Mollari, Flavia Silvia Galli, Maria Teresa Cerasa, Camilla Cuva, Romano Zilli, Alessandro Ubaldi, Maria Teresa Scicluna, Katia Barbaro, Alberto Mantovani, Daniele Marcoccia

**Affiliations:** 1 Istituto Zooprofilattico Sperimentale del Lazio e della Toscana, Via Appia Nuova 1411, 00178 Roma, Italy; marta.mollari-esterno@izslt.it (M.M.); mariateresa.cerasa-esterno@izslt.it (M.T.C.); camilla.cuva@izslt.it (C.C.); romano.zilli@izslt.it (R.Z.); alessandro.ubaldi@izslt.it (A.U.); teresa.scicluna@izslt.it (M.T.S.); katia.barbaro@izslt.it (K.B.); 2 Study Centre KOS–Sciente Art Society, Piazza Gandhi, 3, 00144 Roma, Italy; alberto.mantovani1956@gmail.com

**Keywords:** disinfection by-products (DBPs), steatosis, endocrine disruption, nuclear receptors, lipid metabolism

## Abstract

Disinfection byproducts (DBPs) are ubiquitous contaminants formed during drinking water treatment and are traditionally regulated based on cytotoxic and genotoxic endpoints. However, evidence suggests that DBPs may also act as metabolic disruptors interfering with hepatic metabolic pathways. This study investigates the early metabolic disruption and steatogenic effects of four regulated DBPs, bromoform (BR), bromodichloromethane (BDCM), monochloroacetic acid (MCA), and dichloroacetic acid (DCA), using the human hepatic cell models HepG2 (derived from hepatocellular carcinoma) and HUH7 (derived from hepatoblastoma). Cells were exposed to a broad concentration range (1 pM–100 µM) to capture both sub-cytotoxic and mechanistically informative responses at low, environmentally relevant levels. Effects on lipid and sterol metabolism were assessed through the transcriptional modulation of a panel of nuclear receptors (AHR, PXR, RXR, and LXR) and the sterol regulatory enzyme HMG-CoA reductase (HMGCR) as well as intracellular lipid accumulation; cytotoxicity and oxidative stress endpoints were concurrently evaluated. All DBPs tested induced significant, dose-dependent alterations in nuclear receptor signaling and also promoted lipid accumulation in the low-concentration range and without concurrent cytotoxicity; conversely, oxidative stress responses were limited or absent, and HMGCR emerged as a sensitive target, albeit with different patterns (upregulation by BR and MCA, and downregulation by BDCM and DCA). Relevant substance-specific aspects were also observed for other transcriptional targets, e.g., PXR upregulation was particularly evident for BR and BCDM while DCA downregulated the tested receptors. DBP-induced lipid accumulation was more pronounced in HUH7. Regulated DBPs can elicit early steatogenic and metabolic effects even at concentrations below current regulatory thresholds. The findings highlight that endocrine–metabolic disruption should be considered as a relevant endpoint in DBP risk assessment.

## 1. Introduction

The disinfection of water intended for human consumption using oxidizing agents such as chlorine, chloramines, chlorine dioxide, and ozone is a fundamental practice for ensuring microbiological safety and preventing the transmission of waterborne pathogens [1,2,3]. However, the application of these oxidants inevitably leads to the formation of disinfection byproducts (DBPs), which originate from reactions between disinfectants and natural organic matter (NOM), naturally occurring brominated and iodinated compounds in source waters, and other reactive inorganic species [4,5,6,7]. Among the DBPs of greatest regulatory and toxicological concern are trihalomethanes (THMs), including bromoform (BR) and bromodichloromethane (BDCM), and haloacetic acids (HAAs), such as monochloroacetic acid (MCA) and dichloroacetic acid (DCA) [7,8]. The formation and speciation of these compounds is influenced by multiple factors, including source water quality, the concentration and nature of organic precursors, the presence of naturally occurring halides, pH, temperature, and the operational conditions of the disinfection process [4,7,8]. In particular, waters enriched in humic organic matter or bromide tend to exhibit higher levels of brominated DBPs, which are frequently associated with increased biological reactivity and toxicity [9]. Although advanced treatment processes—such as activated carbon filtration, ozonation followed by biofiltration, and precursor-removal techniques—can substantially reduce DBP formation, their complete elimination remains impracticable without compromising disinfection efficacy [6,10,11]. These compounds are regulated because chronic exposure, even at low concentrations, has been associated with potential adverse effects on the liver, kidneys, and other target organs, as well as possible carcinogenic outcomes reported in animal studies and also by a number of epidemiological investigations [7,12,13,14]. The growing concern regarding DBPs arises from their widespread occurrence throughout drinking water distribution systems and from the intrinsic difficulty of achieving their complete removal without compromising microbiological safety [15]. Consequently, major international regulatory frameworks have established stringent guideline values to limit population exposure [16]. In the United States, the Environmental Protection Agency (U.S. EPA) has set a maximum limit of 80 µg/L for total TTHMs and 60 µg/L for the sum of HAA5 as thresholds intended to balance chemical risk reduction with effective microbiological control [17,18]. Similarly, Directive (EU) 2020/2184 establishes a limit of 60 µg/L for HAAs and requires Member States (MSs) to implement a DBP-minimization strategy, promoting improvements in precursor-removal treatments, optimization of disinfection conditions, and continuous monitoring along the distribution network [11].

Recent monitoring data indicate that disinfection byproducts occur in drinking water at concentrations that typically range between ~1–100 μg/L for trihalomethanes (THMs) and ~1–80 μg/L for haloacetic acids (HAAs), as reported by major international regulatory agencies including the U.S. EPA, the World Health Organization (WHO), and the European Union in recent assessments (2023–2025). These levels represent the exposure ranges commonly found in distribution systems and serve as the basis for maximum contaminant limits established to control chronic population exposure [7,11,16,17,18].

Nevertheless, while the regulatory limits correspond to very low environmental concentrations, toxicological evidence indicates that several DBPs, when evaluated at levels comparable to the highest range of those typically detected in drinking water, are capable of triggering adverse cellular responses in hepatocytes in vitro, such as the induction of oxidative stress and impaired mitochondrial function, which are also consistently associated with structural and functional damage in vivo [12,19]. In addition, limited data suggest that DBPs can alter the expression of 3-hydroxy-3-methylglutaryl-CoA reductase (HMGCR), a key regulatory component of hepatic sterol and lipid metabolism and a transcriptional marker of metabolic disturbances [20]. Indeed, HMGCR transcription is tightly regulated by complex regulatory circuits involving Sterol Regulatory Element-Binding Protein 2 (SREBP-2), Liver X Receptors (LXRs), and feedback mechanisms driven by intracellular cholesterol and isoprenoid levels [21]. Moreover, several DBPs have been shown to modulate the activity of key nuclear receptor-mediated signaling pathways involved in xenobiotic and lipid metabolism, including the aryl hydrocarbon receptor (AHR), the pregnane X receptor (PXR), and the retinoid X receptor (RXR), as well as LXRs. These interactions lend further support to the hypothesis that DBPs may contribute to transcriptional dysregulation and to alterations in lipid homeostasis, processes that are frequently associated with hepatic steatosis and pro-carcinogenic responses [12,22,23]. Consequently, the assessment of HMGCR expression with nuclear receptor analyses can provide a panel of markers to evaluate the metabolic disruption potential of DBPs in the liver. Furthermore, an increasing body of evidence suggests that certain DBPs may act as endocrine-disrupting chemicals (EDCs) [24,25], capable of inducing adverse health effects by interfering with the endogenous hormonal system [26,27,28]. EDCs have mainly been linked to disorders and tumors of the male and female reproductive tracts [27,28,29,30]; furthermore, in the last decade, the association of EDCs with metabolic syndrome aspects (obesity, diabetes, and liver steatosis) has received growing attention [31]. Although DBPs are not yet formally classified as EDCs, the ability of several DBPs to modulate nuclear receptors such as AHR, PXR, RXR and LXR may impact both lipid homeostasis and the hormone regulation of metabolism [12,24]; thus, the potential endocrine-related modes of action of DBPs deserve further investigation using targeted in vitro models [24]. In the present study, we investigated the metabolism-disrupting profile in vitro of a group of DBPs using a representative panel of parameters. The study used a broad concentration range (1 pM–100 µM). In particular, the lower concentration range (pM–nM) encompasses levels comparable to human safety in order to detect early metabolic disturbances in the absence of overt cytotoxicity [18]. The upper concentration range (up to 100 μM) is intended to characterize dose–response relationships of downstream events, including the identification of transcriptional activation thresholds and the analysis of compound-specific signaling patterns. This tiered approach, consistent with current AOP-oriented frameworks for metabolic disruption, provides a comprehensive evaluation of both upstream events and higher-dose downstream responses [17,18].

We compared DBPs in the human hepatic cell models HepG2 and HUH7, two established experimental systems originating from liver tumors of different histological origin, namely, hepatoblastoma (HepG2) and hepatocellular carcinoma (HUH7). The inclusion of HMGCR in the panel of analyzed genes enabled a more comprehensive assessment of whether DBPs can induce early alterations in sterol biosynthesis and regulation and/or contribute to steatogenic patterns [32]. Luminescence-based assays were used as general indicators of cellular metabolic status to evaluate the relationship, under the experimental conditions applied, between the activation of nuclear receptor-mediated pathways and markers of general cell stress such as the increase in reactive oxygen species (ROS) [33]. The DBPs investigated were selected based on previous data suggesting metabolic disruption potential. The results present the patterns of effects elicited by each substance [13,31].

## 2. Materials and Methods

### 2.1. Chemicals

The following chemicals were used in this study: bromoform (BR) (CAS No. 75-25-2, ≥98% purity; Sigma-Aldrich, Munich, Germany); bromodichloromethane (BDCM) (CAS No. 75-27-4, ≥97% purity; Sigma-Aldrich, Munich, Germany), monochloroacetic acid (MCA) (CAS No. 79-11-8, >98% purity; Sigma-Aldrich, Munich, Germany) and dichloroacetic acid (DCA) (CAS No. 79-43-6, ≥99% purity; Sigma-Aldrich, Munich, Germany). All solvents were of high-performance liquid chromatography grade.

### 2.2. Cell Culture and Reagents

Cell culture reagents, such as RPMI1640 without phenol red, fetal bovine serum (FBS), charcoal-stripped fetal bovine serum (CS-FBS), HEPES and phosphate buffer solution (PBS), were purchased from Euroclone (Milan, Italy), and penicillin/streptomycin (P/S), amphotericin B and ethanol were supplied by Sigma-Aldrich. HepG2 cells (a human liver cell line derived from a hepatoblastoma) were obtained from Leibniz Institute DSMZ (German Collection of Microorganisms and Cell Cultures). HUH7 cells, derived from a hepatocellular carcinoma of a Japanese male, were purchased from Cytion GmbH (Heidelberg, Germany). The HepG2 and HUH7 cell lines were grown in complete RPMI1640 medium without phenol red supplemented with 10% FBS, P/S 1%, HEPES and 0.1% amphotericin B. For the Treatment Medium, RPMI1640 without phenol red and 10% CS-FBS were used. Before treatment, HepG2 and HUH7 cells were synchronized overnight in Starvation Medium grown without serum and washed once with 1× PBS pH 7.2 w/o Ca^2+^ and Mg^2+^, treated with BR, BDCM, MCA and DCA. All chemicals were dissolved in ethanol to prepare 100 mM stock solutions, which were used as controls (CTRLs) in all experiments. Experiments were performed in three biological replicates. All cells were maintained in a humidified incubator at 37 °C with 5% CO_2_ concentration.

### 2.3. Cell Viability

HepG2 and HUH7 cells were cultured in complete RPMI 1640 medium without phenol red supplemented with 10% FBS and 1% P/S. Subsequently, cells were detached using trypsin without phenol red, and 10,000 cells per well were seeded in 96-well plates in complete RPMI 1640 medium without phenol red, supplemented with 10% CS-FBS and 1% P/S. The cells were then incubated at 37 °C in a humified atmosphere with 5% CO_2_. Following a 24 h incubation period, cells were treated with the test substances at concentrations ranging from 1 pM to 100 µM for 48 h before the cytotoxicity evaluation. All compounds were dissolved in EtOH to obtain 100 mM stock solutions. Stock solutions and working dilutions were prepared in 1x RPMI1640 without serum and P/S just before use, ensuring a final EtOH concentration of 0.01% (*v*/*v*). Cells treated with only the vehicle (0.01% EtOH) were used as controls (CTRLs) in all experiments, which were conducted in three independent biological replicates. The cytotoxic effects of the study substances were assessed using the MTT assay (3-4,5-dimethylthiazol-2-yl)-2,5-diphenyl tetrazolium bromide) on HepG2 and HUH7. Cells were incubated with MTT reagent at a final concentration of 0.5 mg/mL for 4 h at 37 °C. After incubation, the resulting formazan crystals were solubilized using dimethyl sulfoxide (DMSO) and absorbance was measured at 540 nm using the SpectraMax i3x microplate reader (Molecular Devices, San Jose, CA, USA).

### 2.4. Gene Expression Analysis by Real-Time PCR

The HepG2 and HUH7 cell lines, plated in RPMI 1640 medium without phenol red, supplemented with 10% CS-FBS and 1% P/S at a density of 1.0 × 10^6^ cells, were treated with bromoform, bromodichloromethane, chloroacetic acid and dichloroacetic acid and further incubated for 48 h. Upon cell harvesting, total RNA was extracted and purified using the PureLink RNA Mini kit (Invitrogen, Carlsbad, CA, USA). Purified RNA was reverse-transcribed (5 min at 25 °C, 20 min at 46 °C and 1 min at 95 °C) in a final volume of 20 mL using the “iScript™ cDNA Synthesis kit” (BioRad, Hercules, CA, USA) and the obtained cDNA concentration was assessed using the NanoDrop™ ND-1000 spectrophotometer (Thermo Scientific, Rodano (MI), Italy). Each cDNA (500 ng for reaction, in the presence of 500 nM of each primer; Table 1) was amplified in triplicate by real-time RT-PCR (qPCR) using “SsoAdvancedTM SYBR^®^ Green Supermix” (BioRad) in QuantStudio™ Flex Real-Time PCR System (Applied Biosystems, Foster City, CA, USA) in a 40-cycle run (15 s at 95 °C; 30 s at 60 °C) upon an initial step at 95 °C for 30 s. All calculated quantification cycles (Cq) were less than 30. Results were normalized using Glyceraldehyde-3-Phosphate Dehydrogenase (GAPDH) as a reference gene. An additional melting curve was recorded to confirm the specificity of the reaction.

### 2.5. Oil Red O (ORO) Staining Assay for Lipid Detection in Cell Lines

For the Oil Red O (ORO) staining assay, HepG2 and HUH7 cells were cultured in complete RPMI1640 medium without phenol red, supplemented with 10% FBS and 1% P/S, as described in Section 2.3. Cells were detached using phenol red-free trypsin and seeded at a density of 10,000 cells per well in 96-well plates containing complete RPMI1640 medium without phenol red, supplemented with 10% charcoal-stripped CS-FBS and 1% P/S. Cultures were maintained at 37 °C in a humidified atmosphere with 5% CO_2_. After 24 h, cells were treated with the test compounds at concentrations of 1 pM, 10 nM, and 100 µM for 48 h. At the end of the exposure period, cells were gently rinsed with PBS to minimize detachment and fixed in 4% paraformaldehyde (PFA) per well and incubated for 20 min at room temperature on a level surface without agitation. Cells were then washed with PBS for 5 min and rinsed with 60% isopropyl alcohol (IPA). Subsequently, 100 µL of Oil Red O working solution was added to each well and plates were then incubated at room temperature for 15–30 min. For dye extraction, PBS was removed by aspiration, and 100 µL of IPA was added to each well. Plates were placed on a tilting platform and incubated for 5 min at room temperature. The IPA solution containing the extracted Oil Red O was then transferred to a new plate and centrifuged at 2300× *g* for 1 min at room temperature. The supernatant was transferred to a new plate, and the Oil Red O content was quantified spectrophotometrically at 518 nm using a SpectraMax i3x microplate reader (Molecular Devices).

### 2.6. ROS-Glo™ H_2_O_2_ Assay for Evaluation of Antioxidant Activity in Hepatic Cell Lines

Hydrogen peroxide (H_2_O_2_) production was quantified using the ROS-Glo™ H_2_O_2_ Assay kit (Promega, Madison, WI, USA, TM391) according to the manufacturer’s instructions. HepG2 and HUH7 cells were seeded in 96-well plates at a density of 10,000 cells per well in RPMI1640 medium without phenol red, supplemented with 10% charcoal-stripped fetal bovine serum (CS-FBS) and 1% P/S. After 24 h of attachment, cells were treated with the test compounds at concentrations of 1 pM, 10 nM, and 100 µM for 48 h. For detection, the H_2_O_2_ substrate solution (final concentration 25 µM) was added to each well, and cells were incubated for an additional 6 h at 37 °C in a humidified atmosphere with 5% CO_2_. Subsequently, 100 µL of ROS-Glo™ Detection Solution—prepared by reconstituting the luciferin reagent with d-cysteine and Signal Enhancer—was added to each well. Luminescence was measured after 20 min of incubation at room temperature using a SpectraMax i3x microplate reader (Molecular Devices). Experimental controls included wells containing cells treated with vehicle alone, as well as cell-free wells with and without test compounds, to discriminate between cell-dependent and abiotic H_2_O_2_ production.

### 2.7. Statistical Analysis

All data were obtained from three independent experiments performed in triplicate. Cytotoxicity, lipid accumulation, and antioxidant activity results were expressed as mean ± SD and analyzed using the non-parametric Dunnett test for multiple comparisons (SAS JMP Statistical Discovery v14.0, Milan, Italy). Gene expression data, expressed as arbitrary units after normalization to the GAPDH housekeeping gene, were reported as mean ± SD and analyzed using Student’s *t*-test (SAS JMP Statistical Discovery v14.0, Milan, Italy).

Throughout the study, *p*-values ≤ 0.05 *, ≤0.01 **, and ≤0.001 *** were considered statistically significant, as indicated in the figure legends.

## 3. Results

**Figure 1:**
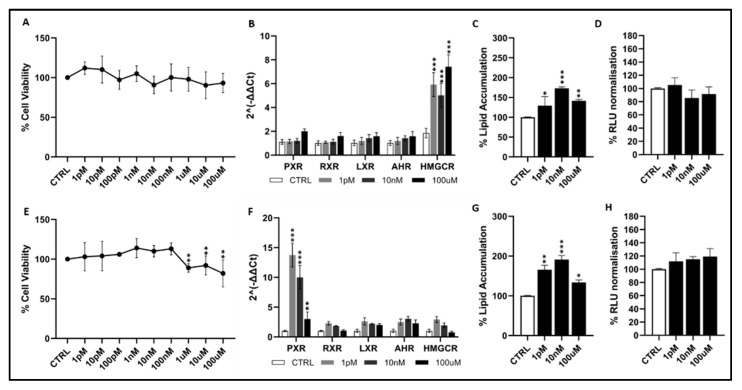
Effects of bromoform on cell viability, gene expression, lipid accumulation, and oxidative stress in HepG2 (**A**–**D**) and HUH7 (**E**–**H**) cells. Gene symbols: *PXR* (Pregnane X Receptor, Gene ID: 8856), *RXR* (Retinoid X Receptor, Gene ID: 6256), *LXR* (Liver X Receptor, Gene ID: 10062), AHR (Aryl Hydrocarbon Receptor, Gene ID: 196), and *HMGCR* (3-Hydroxy-3-methylglutaryl-CoA Reductase, Gene ID: 3156). Normalized RLU values refer to luminescence data normalized to vehicle-treated controls. Data represent mean ± SEM of 3 independent experiments. Statistical significance vs. controls is indicated: *p* < 0.05 *; *p* < 0.01 **; *p* < 0.001 *** (statistical test: *t*-test and Dunnett’s test).

Bromoform: In HepG2 cells (Figure 1A), exposure to BR across the entire concentration range tested (1 pM–100 µM) did not produce detectable cytotoxicity. Cell viability remained stable, consistently ranging between approximately 95% and 110% compared to the control group. In contrast, HUH7 cells (Figure 1E) exhibited a mild but measurable decrease in viability at higher concentrations. Viability started to decline to approximately 85–90% at 10 µM and further to 75–80% at 100 µM, reaching statistical significance. BR triggered distinct transcriptional responses in the two cell lines. In HepG2 (Figure 1B), HMGCR was strongly and dose-dependently upregulated, showing clear induction at 10 pM and reaching maximal levels at 100 µM (approximately 7–8-fold for HMGCR; *p* < 0.001). PXR, RXR and LXR showed small but statistically significant variations (≤1.5–2-fold), detectable mainly at the highest concentrations. In HUH7 cells (Figure 1F), PXR activation was the most pronounced event, with a marked increase even at 1 pM (~10-fold; *p* < 0.001), remaining elevated throughout the concentration range, whereas only slight alterations were observed for AHR, HMGCR, RXR, and LXR. Assessment of intracellular lipid accumulation further supported the steatogenic potential of BR. In HepG2 (Figure 1C), lipid content increased significantly, with a maximal response observed at 10 nM (approximately 40–50%; * *p* < 0.001) and a moderate but still significant increase at 100 µM (approximately 25–30%; *p* < 0.01). No significant effect was detected at 1 pM. In HUH7 cells (Figure 1G), lipid accumulation started at the lowest concentration (1 pM), reached its maximum at 10 nM (~40–50%; *p* < 0.001), and remained significantly increased at 100 µM (~20–25%; *p* < 0.05). Finally, normalized luminescence values (Figure 1D,H) did not differ significantly from their respective controls, indicating that the luminescent endpoint measured was not substantially altered under the conditions tested. Taken together, these results show that BR activates xenobiotic and lipid-related transcriptional pathways while promoting lipid accumulation in both hepatic models. In HepG2 cells, these effects occur without any cytotoxicity, whereas in HUH7 cells, just a mild decrease in viability is observed at the highest concentrations.

**Figure 2:**
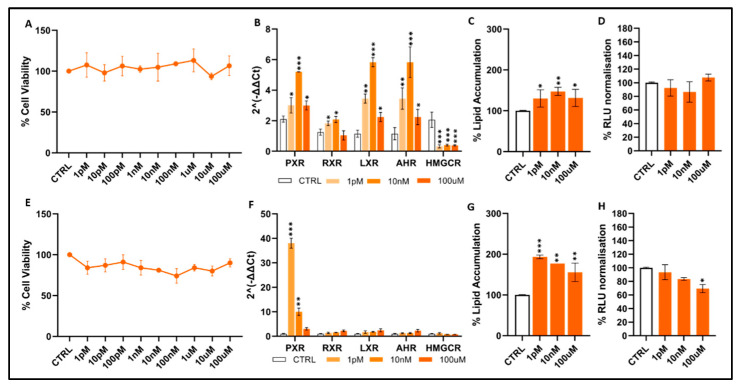
Effects of bromodichloromethane on cell viability, gene expression, lipid accumulation, and oxidative stress in HepG2 (**A**–**D**) and HUH7 (**E**–**H**) cells. Gene symbols: *PXR* (Pregnane X Receptor, Gene ID: 8856), *RXR* (Retinoid X Receptor, Gene ID: 6256), *LXR* (Liver X Receptor, Gene ID: 10062), *AHR* (Aryl Hydrocarbon Receptor, Gene ID: 196), and *HMGCR* (3-Hydroxy-3-methylglutaryl-CoA Reductase, Gene ID: 3156). Normalized RLU values refer to luminescence data normalized to vehicle-treated controls. Data represent mean ± SEM of 3 independent experiments. Statistical significance vs. controls is indicated: *p* < 0.05 *; *p* < 0.01 **; *p* < 0.001 *** (statistical test: *t*-test and Dunnett’s test).

Bromodichloromethane: Exposure to BDCM across the full concentration range (1 pM–100 µM) did not induce measurable cytotoxicity in either HepG2 (Figure 2A) or HUH7 cells (Figure 2E); cell viability consistently ranged from approximately 90% to 110% of the control. Although it did not induce any detectable cytotoxicity, BDCM modulated the expression of several nuclear receptor-related genes, albeit with distinct profiles between the two cell lines. In HepG2 cells (Figure 2B), PXR, RXR, LXR and AHR were significantly upregulated, particularly at 10 nM and 100 µM, reaching approximately 2–3-fold increases relative to the control (*p* < 0.05 to *p* < 0.001). In contrast, HMGCR was significantly downregulated at all tested concentrations in HepG2 cells. In HUH7 cells (Figure 2F), bromodichloromethane elicited a highly selective response, characterized by a marked activation of PXR even at 1 pM (over 20-fold compared to the control; *p* < 0.001). In contrast, RXR, LXR, AHR, and HMGCR remained essentially unchanged at all treatment concentrations. BDCM also increased lipid accumulation in both hepatic models. In HepG2 cells (Figure 2C), intracellular lipid content increased moderately but significantly at 10 nM and 100 µM (~20–30%; *p* < 0.05–0.01); in HUH7 cells (Figure 2G), lipid accumulation increased even at 1 pM, with a particularly marked rise at 10 nM (approximately +60–70%; *p* < 0.001), and its level remained significantly elevated even at 100 µM (approximately +50–60%; *p* < 0.05). These observations indicate that lipid dysregulation represents a consistent and sensitive cellular response to BDCM exposure. Finally, normalized RLU values (Figure 2D,H) showed no major alterations in either cell line, even though HUH7 cells displayed a slight but significant increase at the highest concentration tested (*p* < 0.05). Since the luminescent readout reflects ATP content, reporter activity and general metabolic output, these findings further support that BDCM alters nuclear receptor-associated pathways and promotes lipid accumulation in hepatocyte-derived cells without inducing any general cellular stress.

**Figure 3:**
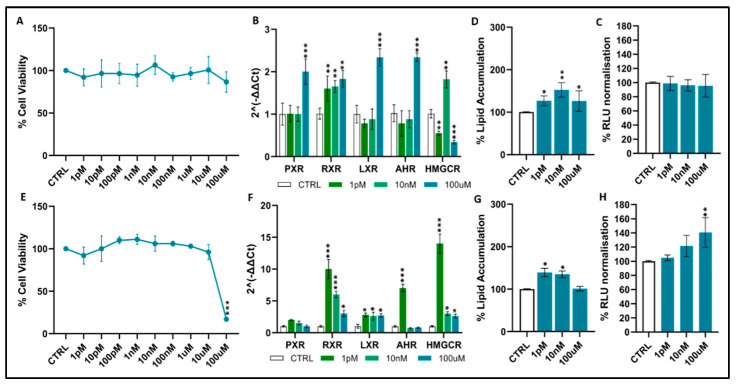
Effects of monochloroacetic acid on cell viability, gene expression, lipid accumulation, and oxidative stress in HepG2 (**A**–**D**) and HUH7 (**E**–**H**) cells. Gene symbols: *PXR* (Pregnane X Receptor, Gene ID: 8856), *RXR* (Retinoid X Receptor, Gene ID: 6256), *LXR* (Liver X Receptor, Gene ID: 10062), *AHR* (Aryl Hydrocarbon Receptor, Gene ID: 196), and *HMGCR* (3-Hydroxy-3-methylglutaryl-CoA Reductase, Gene ID: 3156). Normalized RLU values refer to luminescence data normalized to vehicle-treated controls. Data represent mean ± SEM of 3 independent experiments. Statistical significance vs. controls is indicated: *p* < 0.05 *; *p* < 0.01 **; *p* < 0.001 *** (statistical test: *t*-test and Dunnett’s test).

Monochloroacetic acid: In HepG2 cells (Figure 3A), MCA did not induce detectable cytotoxicity: across the full concentration range tested, cell viability remained stable, ranging from about 90% to 110% compared to controls. In contrast, HUH7 cells (Figure 3E) exhibited a significant decrease in viability at the highest exposure level, 100 µM, with a sharp reduction to approximately 40–50% of the control values. MCA modulated the expression of several xenobiotic- and lipid-responsive genes in both cell lines. In HepG2 cells (Figure 3B), PXR, RXR and LXR were significantly upregulated, with ~2–4-fold increases at 10 nM and 10 µM (*p* < 0.05–0.001). AHR and HMGCR followed a similar trend, showing significant induction at intermediate concentrations (~3–5-fold). At 100 µM, however, expression of several genes—particularly HMGCR—declined. In HUH7 cells (Figure 3F), RXR, LXR, AHR, and HMGCR were significantly upregulated, showing increases ranging from 5- to 10-fold; in contrast, PXR expression was low and remained unchanged up to 10 µM, with a slight reduction only at 100 µM. MCA also increased lipid accumulation in both cell lines. In HepG2 cells (Figure 3D), exposure to 10 nM and 10 µM produced a moderate but significant rise in lipid content (~20–30%; *p* < 0.05–0.01), whereas HUH7 cells (Figure 3G) exhibited a stronger response: lipid accumulation increased by ~80–100% at 10 nM (*p* < 0.001) and remained significantly elevated at 10 µM (*p* < 0.05) as well as at 100 µM, when a concurrent reduction in viability was present. Effects on cellular luminescence were comparatively limited. In HepG2 cells (Figure 3C), normalized RLU values remained unchanged, in parallel with the absence of cytotoxicity. In HUH7 cells (Figure 3H), RLU values increased significantly at 100 µM (*p* < 0.05), consistent with metabolic alterations accompanying cytotoxicity at this dose.

**Figure 4:**
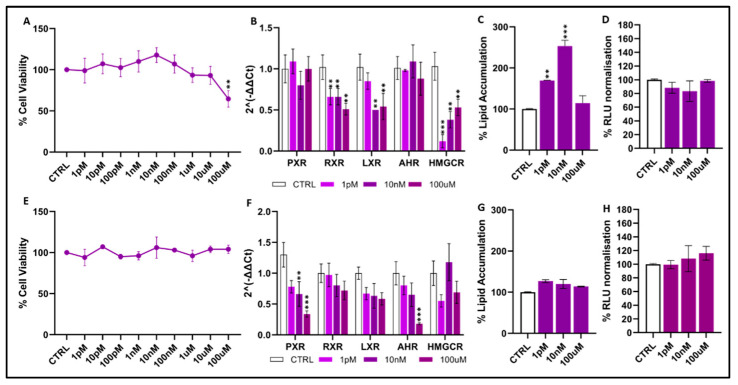
Effects of dichloroacetic acid on cell viability, gene expression, lipid accumulation, and oxidative stress in HepG2 (**A**–**D**) and HUH7 (**E**–**H**) cells. Gene symbols: *PXR* (Pregnane X Receptor, Gene ID: 8856), *RXR* (Retinoid X Receptor, Gene ID: 6256), *LXR* (Liver X Receptor, Gene ID: 10062), *AHR* (Aryl Hydrocarbon Receptor, Gene ID: 196), and *HMGCR* (3-Hydroxy-3-methylglutaryl-CoA Reductase, Gene ID: 3156). Normalized RLU values refer to luminescence data normalized to vehicle-treated controls. Data represent mean ± SEM of 3 independent experiments. Statistical significance vs. controls is indicated: *p* < 0.01 **; *p* < 0.001 *** (statistical test: *t*-test and Dunnett’s test).

Dichloroacetic acid: In HepG2 cells (Figure 4A), DCA did not induce cytotoxicity at low or intermediate concentrations, as cell viability remained comparable to control values (~95–110%): a clear reduction (30–40% compared to control values) was observed only at the highest dose, 100 µM. In HUH7 cells (Figure 4E), it showed no effects on cell viability across all concentrations tested. In terms of transcriptional effects, HepG2 cells (Figure 4B) exhibited relatively modest gene-expression changes, evidenced by slight downregulation of PXR, RXR and LXR at 10 nM and 100 µM, though without a clear dose-dependent pattern. AHR displayed a minor but significant decrease at 100 µM (*p* < 0.05), while HMGCR showed the most pronounced response, with significant reductions at both 10 nM and 100 µM (*p* < 0.01–0.001). Conversely, HUH7 cells (Figure 4F) showed stronger transcriptional repression, with PXR and RXR significantly decreased at 10 nM and 100 µM (*p* < 0.05–0.001), while LXR followed a similar but more moderate trend. Both AHR and HMGCR were also significantly downregulated at the highest concentrations. DCA also affected lipid homeostasis in both models. In HepG2 cells (Figure 4C), exposure to 100 µM led to a significant increase in intracellular lipid accumulation (~30–40%; *p* < 0.05), while 10 nM produced a much stronger rise, approaching 200% of controls (*p* < 0.001). In HUH7 cells (Figure 4G), lipid accumulation showed a moderate increase across all concentrations, consistently indicating that DCA can promote lipid accumulation in hepatic cells. Effects on cellular luminescence were limited. In HepG2 cells (Figure 4D), normalized RLU values remained unchanged across all concentrations; in HUH7 cells (Figure 4H), a significant increase in RLU was observed only at 100 µM (*p* < 0.05).

## 4. Discussion

The hazard assessment of metabolic disruptors is a current major challenge in toxicological risk assessment [34]. The present study provides a comprehensive evaluation of the effects on hepatic metabolism of four regulated disinfection byproducts (DBPs)—BR, BDCM, MCA and DCA—across a broad concentration range, revealing several mechanistic features that extend the current understanding of DBP toxicity. Notably, all tested DBPs induced transcriptional and metabolic alterations at concentrations far below those typically used in conventional toxicological assays, with several responses occurring with environmentally relevant ranges; the alterations occurred generally at lower exposures than, or even in the absence of, cytotoxicity and general cellular stress, suggesting that nuclear receptors and lipid homeostasis are specific DBP targets.

The most relevant effects observed in the present study—namely the modulation of nuclear receptors, the alteration of HMGCR expression, and lipid accumulation—emerge even at picomolar to nanomolar concentrations, well below those inducing cytotoxicity. These concentrations are also comparable to the higher range of environmentally documented exposures. The dose–response profiles in our study are consistent with those typically described for many metabolism-disrupting chemicals, where receptor-mediated low-dose effects are independent of cellular toxicity [31,35,36]. The clear distinction between sub-cytotoxic metabolic effects and high-dose toxic responses reinforces the physiological and environmental relevance of the concentrations used in this study. Early molecular responses at sub-cytotoxic concentrations reflect alterations in receptor-mediated pathways rather than unspecific consequences of cellular stress. Overall, the patterns of metabolic effects elicited by the tested DBPs at sub-cytotoxic concentrations highlight the mechanistic relevance and specificity of the observed low-dose responses.

Overall, these findings indicate that DBPs may act as metabolic disruptors, triggering early molecular responses and low-dose mechanisms that may not be captured by classical toxicity endpoints. A key observation concerns the differential susceptibility of two hepatic models (HepG2 and HUH7 cells). Indeed, they are both derived from cancer but are of different histological origins: hepatocellular carcinoma (derived from mature hepatocytes) for HepG2 and hepatoblastoma (derived from immature hepatocytes) for HUH7. Hence, the observed differences in response to DBPs might reflect a differential vulnerability according to the developing stage of the hepatocyte. HCGMR was a sensitive target for BR and DCA, while BDCM and MCA elicited a more pleiotrophic modulation. PXR was a sensitive target of BR and BDCM in HUH7, while MCA and DCA displayed a more pleiotrophic pattern. Both cell lines showed DBP-induced lipid accumulation; the dose–response curves showed that the effect was more pronounced in HUH7 for BR, BRDCM and DCA, while it appeared stronger in Hepg2 for MCA. The four tested DBPs showed some shared actions and targets as well as substance-specific patterns. Across the DBPs tested, a consistent finding was the modulation of hepatic nuclear receptor networks involved in lipid and sterol regulation and cross-talk with other nuclear receptors (e.g., ERs and RARs) as well as xenobiotic metabolism. BR and BDCM strongly activated PXR, particularly in HUH7, where responses were detectable at picomolar concentrations; HepG2 exhibited a broader transcriptional sensitivity, with concomitant modulation of RXR, LXR and AHR. MCA induced strong activation of RXR, LXR and AHR at low and intermediate doses in both cell lines, whereas DCA primarily produced transcriptional repression.

In this study, the transcriptional panel targeted a set of master regulatory nodes—namely PXR, AHR, RXR, LXR, and HMGCR—exerting upstream control over broad networks regulating lipid and sterol metabolism. Thus, changes in the restricted panel of genes investigated flag early impacts on downstream pathways involved in lipid synthesis, transport, and oxidation.

Importantly, the observed modulation of nuclear receptors provides indirect but mechanistically meaningful indications regarding the activation or repression of entire metabolic cascades [21,22,23,32,36]. For example, changes in PXR or LXR expression are known to impact SREBP-driven transcription, de novo lipogenesis, fatty acid oxidation programs, and lipid trafficking. Likewise, HMGCR, as a rate-limiting enzyme of the mevalonate pathway, reflects alterations not only in sterol biosynthesis but also in broader metabolic processes interconnected through sterol-sensing mechanisms. Additional downstream targets could certainly be explored in future studies; yet, the current dataset provides a coherent mechanistic framework linking nuclear receptor dysregulation with the steatogenic outcomes observed in both hepatic cell models.

Together, these findings reveal that DBPs influence in a compound-specific and cell-specific manner. Notably, the magnitude of PXR activation by BR and BDCM at picomolar concentrations suggests that these halogenated DBPs may function as potent metabolic modulators even at low environmental exposures.

Furthermore, several studies have demonstrated the broad physiological roles of nuclear receptors such as PXR, AHR, RXR, and LXR, which act as central regulators of xenobiotic metabolism, lipid homeostasis, and hormone-dependent metabolic control, thereby indicating links between environmental exposures and metabolic disorders. Their dysregulation has been implicated in hepatosteatosis and metabolic syndrome, supporting the mechanistic relevance of the pathways affected by DBPs in our study [22,23,31].

The literature from pharmacology and hepatocyte biology shows that perturbation of these nuclear receptors can influence key processes such as SREBP activation, fatty acid synthesis, β-oxidation, cholesterol efflux, and hepatic energy metabolism. In this context, the transcriptional alterations observed in our experiments impinge on established metabolic pathways and help explain the steatogenic outcomes detected in both cell models [21,32,33,36].

HMGCR expression emerged as one of the most sensitive and dynamically regulated endpoints, albeit with substance-related patterns. BR produced strong induction, BDCM consistently suppressed expression in HepG2, MCA displayed a biphasic response with activation at low and intermediate doses followed by repression at high concentration, and DCA consistently inhibited HMGCR. These directionally divergent patterns indicate that sterol regulatory pathways represent a central node affected by DBPs and that HMGCR may serve as an early integrative marker of metabolic perturbation. The presence of non-monotonic responses—such as MCA-induced HMGCR activation at 10 nM followed by suppression at 100 µM—further supports the involvement of autoregulated, receptor-mediated pathways typical of endocrine–metabolic disruption. Intracellular lipid accumulation represented another key mechanistic outcome. All DBPs increased lipid levels with consistent concentration-dependent patterns, and in several cases, steatogenic effects were observed even at sub-cytotoxic concentrations. DCA produced the strongest lipid accumulation, reaching approximately 200% of controls in both cell lines at 100 µM, but BR, BDCM and MCA also induced significant increases at environmentally relevant or low-micromolar doses. Importantly, steatosis occurred without a parallel increase in ROS production, indicating that DBP-induced lipid accumulation is not primarily driven by oxidative stress under the present conditions. Instead, the alignment between lipid accumulation and transcriptional changes in nuclear receptors and HMGCR strongly suggests that hepatic lipid dysregulation arises from metabolic and receptor-mediated pathways rather than from generalized cellular stress.

An additional aspect worth considering is the physiological specialization of hepatocytes in xenobiotic detoxification [22,23]. As the liver is the primary organ responsible for the biotransformation and clearance of foreign chemicals and hormones such as thyroid hormones, hepatic cells possess a highly enriched repertoire of nuclear receptors, phase I and phase II metabolizing enzymes, and lipid-handling pathways that collectively coordinate adaptive responses to external stimuli such as chemical exposures [22,23]. This inherent specialization can explain, at least partly, the sensitivity of hepatocyte-derived models, such as HepG2 and HUH7 cells, to the selective activation of metabolic pathways by low DBP concentrations observed in our study [12,21].

Moreover, hepatocytes are equipped to detect, uptake, and neutralize potentially harmful molecules, including DBPs, with the goal of limiting their systemic distribution. Such physiological functions may elicit specific transcriptional or metabolic responses that differ from those of non-hepatic cell types [12,22,31]. This perspective further supports the relevance of using liver-derived cell models to investigate the early events of metabolic disruption. While we acknowledge that in vivo studies would further strengthen and expand the relevance of our findings for the assessment of risks at the organism level, the present work is a mechanistically oriented in vitro investigation consistent with the developing utilization of New Approach Methodologies (NAMs) [17,18,34]. Early transcriptional and metabolic responses, such as those mediated by nuclear receptors and HMGCR, are difficult to evaluate in vivo without large-scale or invasive study designs. In this context, the exploitation of in vitro hepatic models provides a NAM for identifying early molecular and cellular events of metabolic disruption, supported by biologically plausible and robust mechanistic evidence.

## 5. Conclusions

Collectively, these results indicate that regulated DBPs, traditionally evaluated mainly for genotoxic or cytotoxic potential, may act as metabolic disruptors at environmentally relevant concentrations, exerting potent modulation of nuclear receptors, disruption of sterol regulation, and induction of steatogenic responses in cells derived from either mature or immature hepatocyte. The effects observed at low doses—particularly PXR activation and early metabolic markers—suggest that metabolic disruption is an endpoint worth further consideration in DBP risk assessment. Compound- and cell-specific differences further highlight the need for multilayered evaluations to distinguish conserved mechanisms from model-dependent effects. Our findings expand the mechanistic understanding of DBP toxicity by demonstrating that these compounds elicit early transcriptional and metabolic disturbances independently of cytotoxicity or oxidative stress; noticeably, HMGCR features as a potential integrative marker of DBP-induced metabolic perturbation. These insights point to endocrine–metabolic modes of action in characterizing DBP Points of Departure and biomarkers, highlighting the importance of receptor-mediated pathways in the evaluation of DBP safety.

## Figures and Tables

**Table 1:** Sequences of forward and reverse primers for RT-PCR.

Gene Symbol (mRNA)	Primer	Sequence (5′→3′)
Retinoid X Receptor (*RXR*)	Forward	TTCAAGCTCATCGGGGACAC
	Reverse	CTAAGTCATTTGGTGCGGCG
Pregnane X Receptor (*PXR*)	Forward	CTGTGACAAGGCTACGCTGA
	Reverse	AGGCAGGCACTTTCATACCC
Liver X Receptor (*LXR*)	Forward	ATCCGTCCACAAAAGCGGAA
	Reverse	TGTAGTGCGCTCCCTTGATG
3-Hydroxy-3-Methylglutaryl-Coenzyme A Reductase (*HMGCR*)	Forward	GTTTCCAGTCCAGGTCAGGG
	Reverse	TGCATGCTCCTTGAACACCT
Aryl Hydrocarbon Receptor (*AhR*)	Forward	CAACAGCAACAGTCCTTGGC
	Reverse	GCCTGGCAGTACTGGATTGT
Glyceraldehyde-3-Phosphate Dehydrogenase (*GAPDH*)	Forward	AGTCCTTCCACGATACCAAAGT
	Reverse	CATGAGAAGTATGACAACAGCCT

## Data Availability

The original contributions presented in this study are included in the article. Further inquiries can be directed to the corresponding authors.
